# Analysis of Gene Expression in 4,4′-Methylenedianiline-induced Acute Hepatotoxicity

**DOI:** 10.5487/TR.2009.25.2.085

**Published:** 2009-06-01

**Authors:** Jung-Hwa Oh, Hea-Jin Yoon, Jung-Sun Lim, Han-Jin Park, Jae-Woo Cho, Myung-Sang Kwon, Seokjoo Yoon

**Affiliations:** 14 Toxicogenomics Team, Korea Institute of Toxicology, 19 Shinsung-ro, Yuseoung, Daejeon, 305-343 Korea; 24 Clinical Pathology Team, Korea Institute of Toxicology, 19 Shinsung-ro, Yuseoung, Daejeon, 305-343 Korea; 34 Korea Institute of Toxicology, 19 Shinsung-ro, Yuseoung, Daejeon, 305-343 Korea; 44 School of Veterinary Medicine, Kangwon National University, Chuncheon, 200-701 Korea

**Keywords:** 4,4′-Methylenedianiline, Hepatotoxicity, Gene expression profiling

## Abstract

4,4′-Methylenedianiline (MDA) is an aromatic amine that is widely used in the industrial synthetic process. Genotoxic MDA forms DNA adducts in the liver and is known to induce liver damage in human and rats. To elucidate the molecular mechanisms associated with MDA-induced hepatotoxicity, we have identified genes differentially expressed by microarray approach. BALB/c male mice were treated once daily with MDA (20 mg/kg) up to 7 days via intraperitoneal injection (i.p.) and hepatic damages were revealed by histopathological observation and elevation of serum marker enzymes such as AST, ALT, ALP, cholesterol, DBIL, and TBIL. Microarray analysis showed that 952 genes were differentially expressed in the liver of MDA-treated mice and their biological functions and canonical pathways were further analyzed using Ingenuity Pathways Analysis (IPA). Toxicological functional analysis showed that genes related to hepatotoxicity such hyperplasia/hyperproliferation (*Timpl*), necrosis/cell death (*Cd14, Mt1f, Timpl*, and *Pmaipl*), hemorrhaging (*Mt1f*), cholestasis (*Akr1c3, Hpx*, and *Slc10a2*), and inflammation (*Cd14* and *Hpx*) were differentially expressed in MDA-treated group. This gene expression profiling should be useful for elucidating the genetic events associated with aromatic amine-induced hepatotoxicity and for discovering the potential biomarkers for hepatotoxicity.

## INTRODUCTION

4,4'-Methylenedianiline (MDA) is a primary aromatic amine used extensively in the manufacture of polyamides, epoxy resins and polymers in variety of polyurethane industry (Mcqueen and Williams, 1990). MDA is an important intermediate in the production of 4,4'-methylenediphenyl diisocyanate (MDI), and also used in the plastic industries (Zhang *et al.*, 2006). Because of widespread use and large scale production of both MDI and MDA, the potential risk for human exposure and intoxication from MDA is significant. Humans exposed to MDA accidentally or occupationally develop injuries to cholangiocytes with subsequent cholestasis, cholangitis, jaundice, toxic hepatitis with elevated serum liver enzymes (Bastian, 1984; Nichols, 2004). Studies in rats have shown that moderate doses of MDA induce a dose- and time-dependent cholestasis, rapid and severe injury to biliary epithelial cells and moderate hepatotoxicity (Santa Cruz *et al.*, 2007). To date, MDA is known to be genotoxic by forming DNA adducts in the liver and induce the DNA damage in rat or human hepatocytes (Martelli *et al.,* 2002; McQueen and Williams, 1990; Schutze *et al.*, 1995). In addition, *N*-Acetyl MDA has been identified as a major metabolite (in rat and human urine) of MDA or MDI (Robert *et al.*, 1995; Schutze *et al.*, 1995). *N*-Acetyl MDA which is catalyzed by *N*-acetyltransferase 1 (NAT1) and 2 (NAT2) has been known to be an important factor for susceptibility toward MDA-induced hepatotoxicity (Zang *et al.*, 2005). However, little is known about its molecular mechanism of MDA-induced hepatotoxicity, although studies about acute MDA exposure indicated that biliary epithelial cells of the liver and common bile duct are the early site of injury.

Microarray analysis have been applied extensively to elucidate the molecular mechanisms underlying hepatotoxicity (Heijne *et al.*, 2004; Huang *et al.*, 2004; Oh *et al.*, 2007), nephrotoxicity (Oh *et al.*, 2006), and other toxicological phenomena (Oh *et al.*, 2009a, b) and to alternatively assess toxicity (Hamadeh *et al.*, 2002). In the present study, gene expression profiling of MDA-induced hepatotoxicity was analyzed using microarray approach to identify the genes associated with liver injury and understand the mechanism of MDA-induced hepatotoxicity in molecular level. Gene expression profiling was here analyzed in mice after administration of MDA across various time points using 10 K Mouse oligo chip, supported by histopathology observation of the liver and biochemical analysis.

## MATERIALS AND METHODS

***Animal and chemical.*** Methylenedianiline (MDA) was purchased from Sigma, and approximately 9-week-old BALB/c male mice (SLC, Inc., Japan) were kept in a 12-h light/dark cycle, under controlled temperature and humidity for 2 weeks prior to experiment in the animal room. The mice were fed standard food pellets. MDA was dissolved in 30% polyethylene glycol and was administered once daily at 20 mg/kg up to 7 day via intraperitoneal injection (i.p.). Five mice were used in each test group and necropsies were then performed at 1, 3 and 7 day.

***Biochemical analysis.*** Blood was collected from the inferior vena cava. Serum was separated by centrifugation. Alanine aminotransferase (ALT), aspartate aminotransferase (AST), alkaline phosphatase (ALP) activities, cholesterol, direct bilirubin (DBIL), and total bilirubin (TBIL) were determined using an automated clinical chemistry analyzer (Fuji Dri-Chem 3500s, Fujifilm Co., Japan), at each time point. Data are expressed as the mean ± S.D. of 3 selected samples with hiotopathological lesion. The statistical significance between various experimental groups was tested using one-way ANOVA followed by the Dunnett test. *P*-values less than 0.05 were considered to be significant.

***Histopathology.*** Liver samples collected from treated and control mice were fixed in 10% neutral buffered formalin, and embedded in paraffin. The sections (4 μm thick) were cut using RM2165 microtome (Leica, Germany), stained with hematoxylin and eosin (H&E), and examined under a light microscope (Nikon E400, Nikon, Japan).

***Isolation of total RNA.*** The left lateral lobe of the liver was removed and processed for RNA extraction. For microarray analysis, total RNA was extracted using the TRIzol reagent (Invitrogen, USA) according to the instructions of the manufacturer and purified using RNeasy total RNA isolation kit (QIAGEN, Germany). Total RNA was quantified by NanoDrop ND-1000 (NanoDrop, USA) and its integrity was assessed by 2100 Bioanalyzer (Agilent Technologies, USA).

***Microarray analysis.*** For microarray analysis, fluorescent-labeled cDNA was prepared by reverse-transcription of total RNA in the presence of the coupled Cy3-dUTP or Cy5-dUTP (NEN) using Superscript II (Invitrogen, USA). Single-stranded cDNA probes were purified using a PCR purification kit (QIAGEN, Germany). Probes were re-suspended in hybridization solution (50% formamide, 5 × SSC, 0.1% SDS). The 10K Mouse oligo chip (GenomicTree, Korea) was hybridized with the fluorescent-labeled cDNAs at 42°C in a humid chamber for 16 hours. After proper washing, the slides were scanned using Axon GenePix 4000B (Axon Instrument, USA). Scanned images were calculated with GenePix 3.0 software (Axon Instrument, USA) and analyzed with Genplex ver. 2.0 software (Istech, Korea). Background of spot intensity was corrected and miscellaneous spot were removed. The log gene expression ratios were normalized by Block-wise centering (Print-tip Lowess normalization) method. The differentially expressed genes were selected based on 2-fold changes in all of the treated groups compared with the levels in the pooled controls on each time point (1, 3, and 7 days). Totally, 952 differentially expression genes were selected and then clustering analysis were performed using *k*-means partitioning clustering based on Euclidean distances ($k = 6$). The biofunctional and canonical pathway analysis of differentially expressed genes was performed using Ingenuity Pathways Analysis (IPA). Selected genes were annotated based on Probe ID with NCBI Genbank accession number.

## RESULTS

***Biochemical evaluation.*** The enzyme activities relative to liver injuries were significantly changed in all time points between MDA-treated and control group (Fig. 1). After 20mg/kg/day MDA treatment, AST which is the index of acute phase liver toxicity was elevated most in day 1 and chronic phase liver enzyme ALT was markedly increased in day 7. ALP and cholesterol activities were also increased in day 3 and day 7. Serum level of direct bilirubin (DBIL) and total bilirubin (TBIL) were all increased in MDA-treated group compared to control group but the value of treated group decreased time dependent manner.

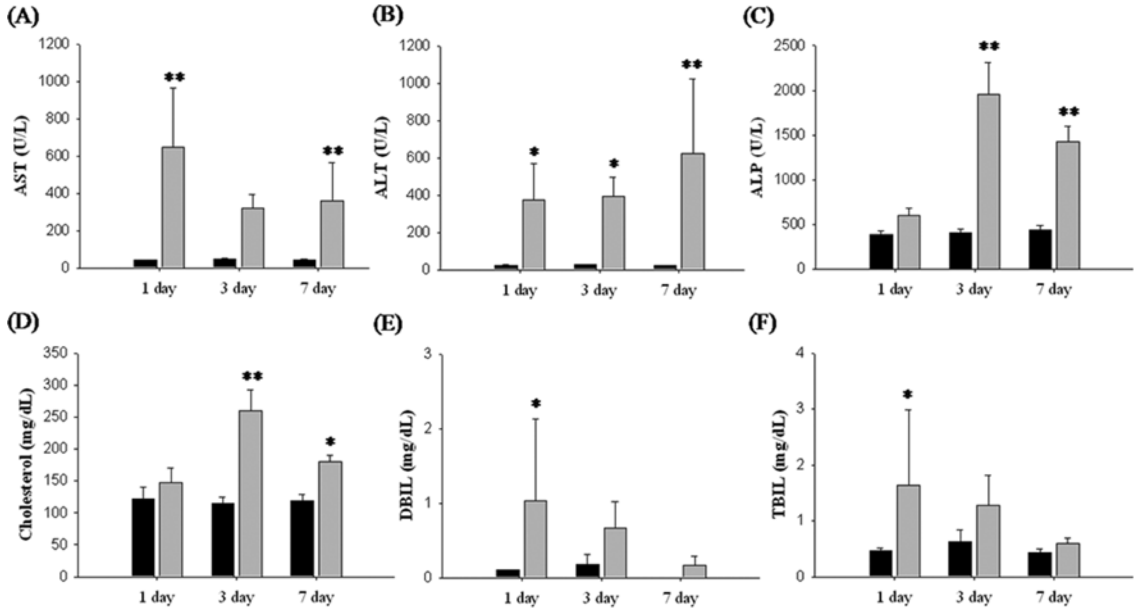

**Fig. 1.** Blood biochemical data in the liver of MDA treated mice. (A) AST (aspartate aminotransferase); (B) ALT (alanine aminotransferase); (C) ALP (alkaline phosphatase); (D) Cholesterol; (E) DBIL (direct bilirubin); (F) TBIL (total bilirubin). Values represent the means ± standard deviation (SD); n = 3 (*$P < 0.05$; ** $P < 0.01$).

**Table 1.** Summary of histophathological lesions in the liver

| Liver pathology | Control (n=5) | MDA-treated group | | |
|---|---|---|---|---|
| | | 1 Day (n = 3) | 3 Day (n = 3) | 7 Day (n = 3) |
| Inflammatory foci | + (3) | | | + (2) |
| Periductular inflammation | | +(3) | ++ (2) +++(1) | ++(2) +++(1) |
| Necrosis/congestion, hepatocyte | | +(1) ++ (1) +++(1) | ++ (2) +++(1) | ++(1) +++ (2) |
| Mitosis | | | ++ (1) | +(1) |
| Ductular hyperplasia | | | | ++(2) |
| Hepatocellular vacuolation, centrilobular | | | +(1) | |
| Regenerative inflammation | | | | ++(1) |

The number of animal with corresponding lesion was represented in the parentheses.
Severity of lesion was indicated as follows; +, mild; ++, medium; +++, severe.

***Histopathology.*** Hepatocytes near the central vein regions of MDA treated mice were compared. Light microscopy showed a decreased in the number of hepatocyte and increased in the size compared to the central vein region of control mice. Periductular inflammation and necrosis/congestion of the hepatocytes were observed in all time points along with the severity of the lesions increased. MDA treatment for 7 days resulted in medium ductular hyperplasia and regenerative inflammation (Table 1).

***Gene expression profiles of MDA-induced hepatotoxicity.*** Microarray analysis was carried out to elucidate the molecular mechanism of hepatic gene expression between vehicle- (30% polyethylene glycol) and MDA-treated mice. Differentially expressed genes in the MDA-treated group were selected based on changes greater than 2-fold compared with the corresponding controls, in all independent experiment. Totally, 952 genes were differentially expressed, including 149, 454, and 577 genes at day 1, day 3, and day 7 in MDA-treated group. To analyze the gene expression patterns according to each time points during MDA-induced hepatotoxicity, total 952 genes were processed in clustering analysis using *k*-means partitioning clustering (Fig. 2). Clustering analysis showed that many genes were down-regulated in the MDA-treated group although individual variation was shown. In particular, many genes belonged to down-regulated clusters were up-regulated at day 3. However, we focused on commonly up- or down-regulated genes in MDA-treated group such as Cluster A and B, respectively. In all time points,

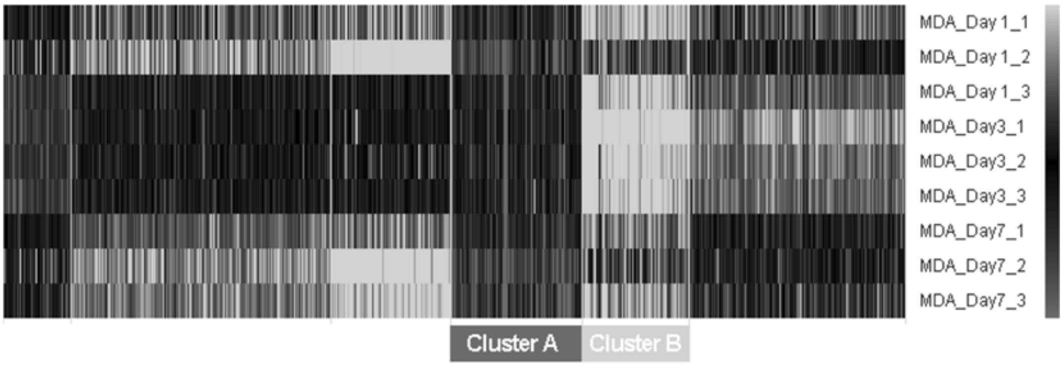

**Fig. 2.** The *k*-means clustering of differentially expressed genes in the liver of MDA treated mice. 952 genes were analyzed based on 2-fold changes and we performed *k*-means clustering by setting *k* = 6 (total clusters = 6). The six clusters are highlighted with colored lines. Cluster A and cluster B indicates the commonly up- or down-regulated group at all time points.

140 genes were significantly up-regulated, while 112 genes were down-regulated in MDA-treated group.

From among the genes up- or down-regulated in common, the top 20 genes that were significantly altered in MDA-treated group were selected and are listed in Table 2 and Table 3. Genes encoding growth factor such as *Gdf15* and *Gdnf*, and several genes encoding various transporters or receptors such as *Fcer1g*, *C3ar1*, *Pgm1*, *Sftpd*, and *Saa3* were up-regulated. In addition, genes related to inflammatory response (*Ccl24* and *Itih3*), regulation of transcription or proliferation (*Hic1* and *Slfn1*), signal transduction (*Apbb1ip* and *Ltb4r1*), and leukocyte chemotaxis or cytoskeleton reorganization (*S100a9*) were up-regulated. On the other hand, genes such as *Pramef12* and *Pr15a8* involved in hormone activity, and ion channel or transport such as *Kcmf1*, *5330439B14Rik*, and *Slc4a4*, were down-regulated. In addition, many genes related to metabolic process (*Plb1*, *Dgat1*, *Naaa*, *Gk2*, and *Dhah*) were down-regulated.

***Toxicological interpretation of differentially expressed genes during MDA-induced hepatotoxicity.*** To investigate the molecular mechanism related to

**Table 2.** Genes up-regulated in the liver of MDA-treated mice

| Gene symbol | Gene title | Acc. no. | Fold Change (Log2) | | |
|---|---|---|---|---|---|
| | | | Day 1 | Day 3 | Day 7 |
| *Gdf15* | growth differentiation factor 15 | NM_011315 | 0.60 | 3.02 | 4.33 |
| *Flot2* | flotillin 2 | L47696 | 3.86 | 5.11 | 4.30 |
| *Fcer1g* | Fc receptor, IgE, high affinity I, gamma polypeptide | NM_009265 | 1.16 | 1.57 | 4.13 |
| *C3ar1* | complement component 3a receptor 1 | NM_009114 | 3.83 | 1.34 | 3.94 |
| *Ccl24* | chemokine (C-C motif) ligand 24 | NM_016701 | 2.31 | 2.21 | 3.92 |
| *Pgm1* | phosphoglucomutase 1 | AK002981 | 2.89 | 4.36 | 3.76 |
| *Sftpd* | surfactant associated protein D | NM_008605 | 2.25 | 3.26 | 3.38 |
| *Hic1* | hypermethylated in cancer 1 | NM_009705 | 1.73 | 0.06 | 3.26 |
| *Slfn1* | schlafen 1 | NM_010766 | 0.41 | 1.53 | 3.19 |
| *Apbb1ip* | amyloid beta (A4) precursor protein-binding, family B, member 1 interacting protein | AK018561 | 1.93 | 1.17 | 3.03 |
| *Gdnf* | glial cell line derived neurotrophic factor | BC003991 | 1.18 | 1.62 | 2.89 |
| *Cd14* | CD14 antigen | NM_009160 | 1.42 | 0.73 | 2.87 |
| *Itih3* | inter-alpha trypsin inhibitor, heavy chain 3 | NM_007737 | 1.07 | 2.03 | 2.76 |
| *Yif1b* | Yip1 interacting factor homolog B (S. cerevisiae) | AK007508 | 2.20 | 0.81 | 2.74 |
| *Ltb4r1* | leukotriene B4 receptor 1 | NM_007743 | 0.73 | 1.62 | 2.39 |
| *Mrps28* | mitochondrial ribosomal protein S28 | AK010014 | 1.67 | 3.17 | 2.38 |
| *S100a9* | S100 calcium binding protein A9 (calgranulin B) | NM_008567 | 2.99 | 4.46 | 2.37 |
| *Cdkl2* | cyclin-dependent kinase-like 2 (CDC2-related kinase) | NM_013710 | 0.98 | 0.36 | 2.31 |
| *Saa3* | serum amyloid A 3 | NM_010662 | 2.06 | 2.22 | 2.31 |
| *Ms4a4b* | membrane-spanning 4-domains, subfamily A, member 4B | NM_023380 | 1.26 | 0.42 | 2.28 |

The fold change was calculated as the relative average value of three arrays in each group compared to controls, and the values are reported as the natural logarithm.

**Table 3.** Genes down-regulated in the liver of MDA treated mice

| Gene symbol | Gene title | Acc. no. | Fold Change (Log2) | | |
|---|---|---|---|---|---|
| | | | Day 1 | Day 3 | Day 7 |
| *Pramef12* | PRAME family member 12 | AK021319 | -3.52 | -4.79 | -4.32 |
| *Kcmf1* | potassium channel modulatory factor 1 | NM_019715 | -2.97 | -3.96 | -2.95 |
| *1700067C01Rik* | RIKEN cDNA 1700067C01 gene | AK006911 | -3.31 | -4.75 | -2.89 |
| *Phb* | prohibitin | AF061744 | -2.78 | -3.91 | -2.80 |
| *5330439B14Rik* | RIKEN cDNA 5330439B14 gene | AK019938 | -2.28 | -2.70 | -2.73 |
| *Pln* | phospholamban | NM_023129 | -3.31 | -3.58 | -2.66 |
| *Plb1* | phospholipase B1 | AK019494 | -3.03 | -4.13 | -2.63 |
| *Slc4a4* | solute carrier family 4 (anion exchanger), member 4 | AF141934 | -2.59 | -3.57 | -2.59 |
| *Dgat1* | diacylglycerol O-acyltransferase 1 | NM_010046 | -2.11 | -2.70 | -2.50 |
| *4921521F21Rik* | RIKEN cDNA 4921521F21 gene | NM_027582 | -2.34 | -3.10 | -2.50 |
| *Sfrp5* | secreted frizzled-related sequence protein 5 | NM_018780 | -3.04 | -4.01 | -2.42 |
| *Tnfsf8* | tumor necrosis factor (ligand) superfamily, member 8 | NM_009403 | -2.74 | -4.25 | -2.41 |
| *Sepw1* | selenoprotein W, muscle 1 | NM_007770 | -2.67 | -3.89 | -2.38 |
| *Prl8a8* | prolactin family 8, subfamily a, member 81 | AK005568 | -2.59 | -3.28 | -2.32 |
| *Gabra3* | gamma-aminobutyric acid (GABA-A) receptor, subunit alpha 3 | NM_008067 | -2.53 | -3.44 | -2.25 |
| *Zfp354c* | zinc finger protein 354C | NM_013922 | -2.33 | -3.81 | -2.22 |
| *Tex18* | testis expressed gene 18 | NM_031385 | -2.99 | -3.65 | -2.21 |
| *Naaa* | N-acylethanolamine acid amidase | AK008776 | -1.88 | -2.63 | -2.18 |
| *Gk2* | glycerol kinase 2 | NM_010294 | -2.26 | -2.92 | -2.18 |
| *Dhdh* | dihydrodiol dehydrogenase (dimeric) | AK005050 | -2.80 | -2.07 | -2.00 |

The fold change was calculated as the relative average value of three arrays in each group compared to controls, and the values are reported as the natural logarithm.

MDA-induced hepatotoxicity, canonical pathways and toxicological pathways integrating differentially expressed genes were analyzed using IPA. Total 252 commonly deregulated genes at all time points including 140 up-regulated and 112 down-regulated genes were characterized. As shown in Fig. 3A, down-regulated genes involved in GABA receptor signaling (*Gabra1, Gabra2*, and *Gabra3*), C21-steroid hormone metabolism (*Akr1c3* and *Hsd3b4*) were identified. Meanwhile, expression of genes related to hepatic fibrosis/hepatic stellate cell activation (*Ccr7*, *Col1a2*, *Cd14*, and *Timp1* for up-regulated, *Fgf1d* for down-regulated), leukocyte extravasation signaling (*Arhgap12*, *Cldn7*, *Itgam*, *Mmp12*, and *Timp1* for up-regulated; *Jam2* for down-regulated), eicosanoid signaling (*Akrk3* and *Ptger3* for up-regulated; *Ltb3r* for down-regulated), and glycosaminoglycan degradation (*Glb1l*, *Hexb* for up-regulated; *Hyal4* for down-regulated) was altered.

The toxicological pathway analysis of differentially expressed genes showed that several toxic mechanism were identified and top ranked mechanisms were selected as shown Fig. 3B. Genes related to positive acute phase response proteins (*Hpx* and *Saa1*), hepatic stellate cell activation (*Cd14* and *Timp1*), negative acute phase response proteins (*Itih3*), cholesterol biosynthesis (*Sqle*), LXR/RXR activation (*Arg2* and *Cd14*) were up-regulated, while genes associated with cytochrome P450 panel (*Cyp4a14*), and anti-apoptosis (*Bcl2l10*) were down-regulated. In addition, genes related to hepatic fibrosis (*Col1as*, *Sparc*, and *Timp1* for up-regulated; *Fgf1* for down-regulated), FXR/RXR activation (*Pck2* and *Slc10a2* for up-regulated; *C6pc2* for down-regulated) were identified. Functional analysis using IPA also identified genes involved in hepatotoxicity such as liver hyperplasia/hyperproliferation, liver necrosis/cell death, liver hemorrhaging, liver cholestasis, and liver inflammation.

## DISCUSSION

Exposure to MDA has been known to induce liver damages and an industrial outbreak of occupational MDA exposure provokes the public health concerns. Using toxicogenomic approaches, toxicological mechanisms and drug safety have been recently investigated. Several studies have shown that the prediction of toxicity is possible by using toxicogenomics, especially hepatotoxicity. To evaluate the molecular mechanisms of acute hepatotoxicity and to identify the molecular targets participating in these processes, we examined the effects of exposure to MDA on gene expression changes in the mice liver.

In the present study, histopathological observation of MDA-treated mice showed that severe hepatic lesions were detected in all time points such as necrosis of hepatocytes. In addition, blood biochemistry showed

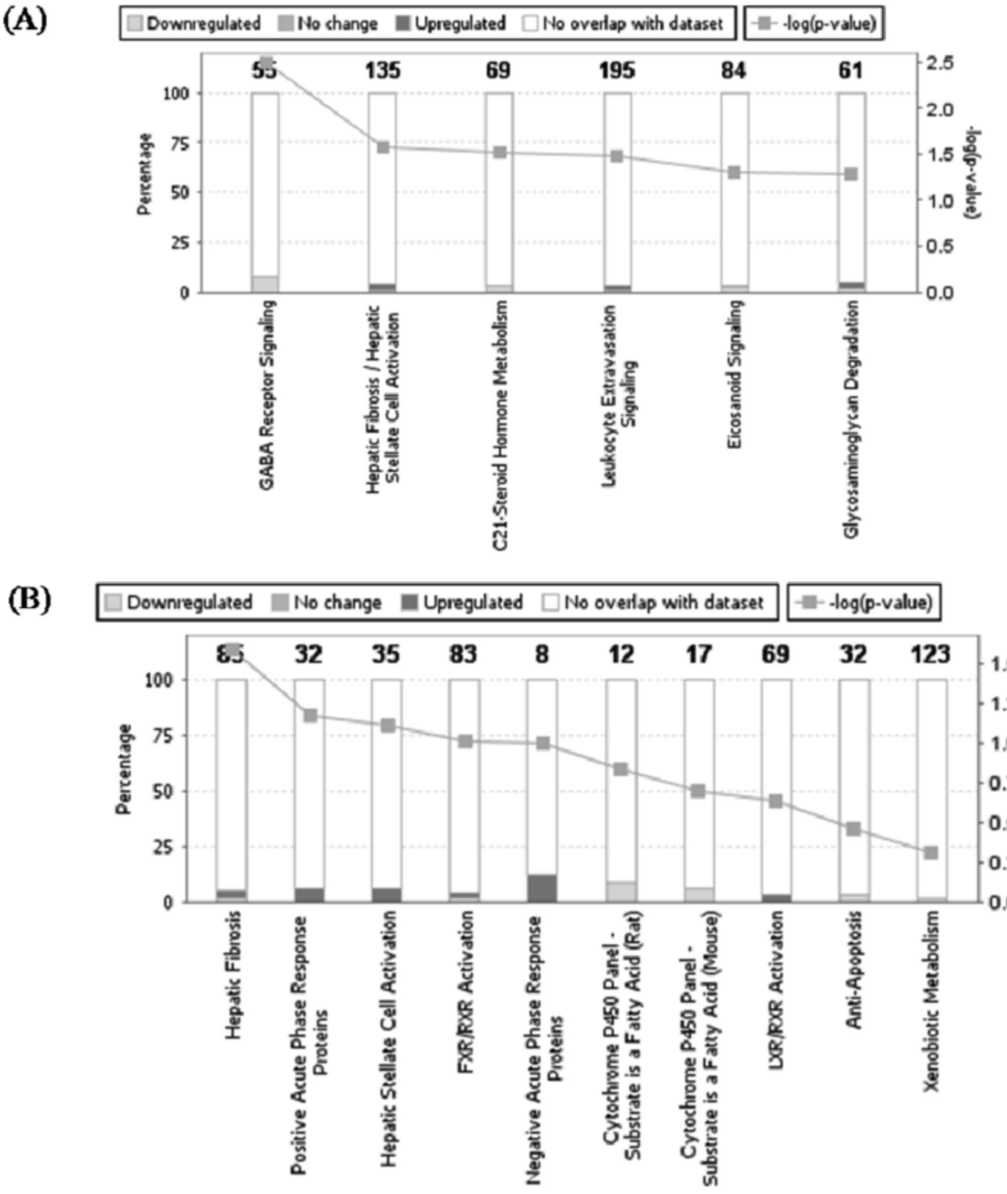

**Fig. 3.** Canonical pathway and toxicological analysis of 252 genes deregulated in MDA-treated group. (A) Canonical pathways which are identified based on statistical significance ($P < 0.05$). (B) Functional analysis showing categories of interest. Arabic numbers upon histogram indicates total number of genes belonged to designated pathways and colored box indicated up- or down-regulated genes which were identified in MDA-treated group as follows; red ones, up-regulated; green ones; down-regulated. Orange line indicates the statistical significance calculated by IPA algorithm, and values means natural logarithm (Log10).

that AST and ALT were significantly increased over at least 7-fold or 15-fold comparing to controls. These results indicate that MDA-induced toxicity has not occurred in a time-dependent manner however severe acute hepatotoxicity was shown in all time points after MDA administrations. Based on *k*-means clustering, microarray analysis also represented that gene expression profiles were not shown in a time-dependent manner: gradually up-regulated or gradually down-regulated in MDA-treated group according to each time point. For this reason we focused on commonly deregulated genes in MDA-treated group and we postulated that significantly up- or down-regulated genes could be potential genomic biomarkers for acute hepatotoxicity.

However, microarray analysis using 10 K mouse oligo chip showed that individual variations among treated group were observed in *k*-means clustering analysis, although liver samples were selected from mice with similar histopathological lesions among MDA-treated group. Quality assessment of microarray experiment using box plot and scatter plot represented that there was no significant variation within an array and between arrays (data not shown). Individual variations might be caused by differences of biological or genetic sensitivity for MDA, although common histopathological lesions were shown. The 10 K mouse oligo chip analysis detected differentially expressed gene sets in MDA-treated group. Among the genes regulated in common at all time points, several genes related to hepatotoxicity including liver hyperplasia/hyperproliferation (*Timp1*), liver necrosis/cell death (*Cd14*, *Mt1f*, *Timp1*, and *Pmap1*), liver hemorrhaging (*Mt1f*), liver cholestasis (*Akr1c3*, *Hpx*, and *Slc10a2*), and liver inflammations (*Cd14* and *Hpx*) were identified using IPA with knowledge-based database. Up-regulation of *Timp1* involved in many diseases, including hepatic fibrosis, aortic stenosis, various cancers, and Alzheimer's disease, were identified in MDA-treated group. *Timp1* belongs to TIMP gene family which is natural inhibitor of the matrix metalloproteinase (MMPs), is known to promote cell proliferation (Gasson *et al*., 1985) and also have an anti-apoptotic function (Liu *et al*., 2005). Regarding hepatotoxicity, it has been known that expression of *Timp1* and MMP promotes the liver fibrosis (Fiorucci *et al*., 2005). In addition, overexpression of *Timp1* is known to block the proliferation (Martin *et al*., 1999) and have anti-apoptotic function in hepatic stellate cells (Murphy *et al*., 2002). Histopathological observation showed that MDA administration induced severe necrosis of hepatocytes and several genes related to liver necrosis such as *Cd14*, *Mt1f*, and *Pmaip1* as well as *Timp1*. *Cd14* is a surface protein preferentially expressed on monocytes or macrophages and its apoptotic function in the liver has been reported (Yin *et al*., 2001). *Mt1f* has been known to involve in apoptosis, oxidative stress, and cytotoxicity and its necrotic function in liver has been also reported (Masters *et al*., 1994). *Pmaip1* containing Bcl2-homology 3 domain has been known to involve in p53 signaling pathway and plays a role in cytotoxicity and apoptosis in various cell type and conditions (Shibue *et al*., 2003; Strohecker *et al*., 2008; Zhang *et al*., 2008). In present study, regarding necrosis or apoptosis of hepatocytes, *Timp1*, *Cd14*, and *Mtlf* were up-regulated, while *Pmaip1* was down-regulated in MDA-treated group. On the other hands, several genes related to cholestasis such as *Akr1c3*, *Hpx*, and *Slc10a2* was also identified. *Akr1c3*, *Hpx*, and *Slc10a2* have been reported that gene expressions were altered during familiar intrahepatic cholestasis, type 1 or type 2 (Alvarez *et al*., 2004; Demeilliers *et al*., 2006). *Slc10a2* also plays a role in transporting the bile acid or salts (Dawson *et al*., 2003) and altered expression of *Slc10a2* suggested that transport might be disrupted. In addition to toxicological function, pathway analysis represented that significant pathways such as GABA receptor signaling or hepatic fibrosis or hepatic cell activation were involved in MDA-induced hepatotoxicity. Our data showed potential biomarkers which are mediated after liver damage. The identified genes offer valuable information related to molecular mechanisms of hepatotoxicity. This data may contribute in elucidation of the molecular events during liver injury and in discovery of the potential biomarkers for hepatotoxicity. To confirm and verify the potential biomarkers for hepatotoxicity, extended microarray analyses using different hepatotoxicants including aromatic amine species are necessary. Furthermore, gene expression comparison in the liver after administration of MDA and active metabolite, N-Acetyl MDA, should be necessary to understand the molecular mechanism of MDA-induced hepatotoxicity.

## ACKNOWLEDGEMENTS

This work was supported by the Ministry of Science and Technology as part of the 2005 Project for Developing a Toxicological Evaluation System for Specific Toxicity-inducing Chemicals at the Korea Institute of Toxicology.
